# Major comorbid conditions in asthma and association with asthma-related hospitalizations and emergency department admissions in adults: results from the German national health telephone interview survey (GEDA) 2010

**DOI:** 10.1186/1471-2466-13-46

**Published:** 2013-07-12

**Authors:** Henriette Steppuhn, Ute Langen, Christa Scheidt-Nave, Thomas Keil

**Affiliations:** 1 Department of Epidemiology and Health Monitoring, Robert Koch Institute Berlin, General-Pape-Strasse 62-66, D-12101 Berlin, Germany; 2 Institute for Social Medicine, Epidemiology and Health Economics, Charité - Universitätsmedizin Berlin, Luisenstrasse 57, D-10117 Berlin, Germany; 3 Institute for Clinical Epidemiology and Biometry, University of Würzburg, Joseph-Schneider-Str. 2, D-97070 Würzburg, Germany

**Keywords:** Acetylsalicylic acid exacerbated respiratory disease, Adult, Allergic rhinitis, Aspirin-induced asthma, Asthma, Gastroesophageal reflux disease, Gastroesophageal reflux, Hospitalization, National health survey

## Abstract

**Background:**

It remains unclear to what extent asthma in adults is linked to allergic rhinitis (AR), gastroesophageal reflux disease (GERD), and acetylsalicylic acid exacerbated respiratory disease (AERD), and how these comorbidities may affect asthma outcomes in the general population. We therefore aimed to assess the prevalence of these major comorbidities among adults with asthma and examine their impact on asthma exacerbations requiring hospital care.

**Methods:**

A total of 22,050 adults 18 years and older were surveyed in the German National Health Telephone Interview Survey (GEDA) 2010 using a highly standardized computer-assisted interview technique. The study population comprised participants with self-reported physician-diagnosed asthma, among which the current (last 12 months) prevalence of AR and GERD-like symptoms (GERS), and life-time prevalence of AERD was estimated. Weighted bivariate analyses and logistic regression models were applied to assess the association of each comorbid condition with the asthma outcome (any self-reported asthma-related hospitalization and/or emergency department (ED) admission in the past year).

**Results:**

Out of 1,136 adults with asthma, 49.6% had GERS and 42.3% had AR within the past 12 months; 14.0% met the criteria of AERD, and 75.7% had at least one out of the three conditions. Overall, the prevalence of at least one exacerbation requiring emergency room or hospital admission within the past year was 9.0%. Exacerbation prevalence was higher among participants with comorbidities than among those without (9.8% vs. 8.2% for GERS; 11.2% vs. 7.6% for AR, and 22.2% vs. 7.0% for AERD), but only differences in association with AERD were statistically significant. A strong association between asthma exacerbation and AERD persisted in multivariable logistic regression analyses adjusting for sex, age group, level of body mass index, smoking status, educational attainment, and duration of asthma: odds ratio (OR) = 4.5, 95% confidence interval (CI) = 2.5–8.2.

**Conclusions:**

Data from this large nation-wide study provide evidence that GERS, AR and AERD are all common comorbidities among adults with asthma. Our data underline the public health and clinical impact of asthma with complicating AERD, contributing considerably to disease-specific hospitalization and/or ED admission in a defined asthma population, and emphasize the importance of its recognition in asthma care.

## Background

Asthma is a common disease among adults [1,2]. It can have a substantial impact on the health-related quality of life and constitutes a high socioeconomic burden on the health care system [2,3]. Allergic rhinitis (AR), gastroesophageal reflux disease (GERD), and acetylsalicylic acid (ASA) intolerance can be assigned to comorbid conditions that are commonly related to asthma [4]. These comorbidities are considered highly relevant for asthma control and exacerbation outcomes [5]. The consideration and specific management of these comorbid conditions is therefore of high importance in asthma care [6].

Allergic rhinitis and asthma commonly coexist [7,8]. Allergic rhinitis is a risk factor for asthma and may even be on the same disease continuum as asthma [7,9,10]. The presence of concomitant AR in adult asthma was associated with worse asthma outcomes resulting in a higher risk of asthma-related emergency hospital care in some studies [7,11,12]. The evaluation and consequent management of AR may improve asthma outcomes according to national and supranational guidelines [6,13,14].

Although an association between gastroesophageal reflux disease (GERD) and asthma has also been reported, the extent and underlying mechanism of this relationship remain unclear [15-17]. There is controversy regarding the effect of GERD on asthma outcomes [5,6,17]. For individuals with asthma and concomitant GERD-like symptoms prior studies only inconsistently suggested that treatment of GERD might reduce exacerbations [17,18].

Intolerance to ASA or other nonsteroidal anti-inflammatory drugs (NSAIDs) marks a distinct clinical entity – ASA exacerbated respiratory disease (AERD), or ASA-induced asthma (AIA) [19-21]. On the basis of intractable inflammation of the upper and lower airways, asthma might precede the development of an upper airway disease comprised of chronic hypertrophic eosinophilic sinusitis and nasal polyps or follow it [19,21]. AERD is characterized by a potentially more severe disease course of asthma [20,22] with a higher likelihood of an asthma-related emergency department or hospital admission [22-24]. Its recognition and appropriate management is crucial to effectively reduce morbidity and acute severe outcomes [6,21].

AR, GERD and AERD are common conditions among adults with asthma. To date, however, prevalence estimates of these conditions among adults with asthma vary widely [16,25,26]. Furthermore, population-based information on the impact of each condition on asthma outcome is scarce, especially with regard to exacerbations that considerably increase morbidity and health care needs [27]. Against this background, we aimed to assess population-based data on the prevalence of AR, GERS and AERD among adults with asthma and examine the association of these comorbidities with asthma exacerbations requiring hospital care in the past 12 months in the framework of a national health interview survey.

## Methods

### Study design and study population

GEDA (“German Health Update”) is a periodically repeated national health interview survey of adults (18 years and older) as part of the continuous health monitoring system in Germany [28]. The target population for the 2010 GEDA survey comprised all adults residing in private households with landline telephones who could fluently speak and understand German language. A two-stage sampling procedure was applied. A random digit dialing method was used for sampling at the household level [29]. At the individual level, the ‘last-birthday-method’ was applied in order to select those adult members of the contacted households who most recently had their birthday prior to the date of the first contact to the respective household [30].

As the participation in the National Health Telephone Interview Surveys is voluntary, at no costs to the survey participants, and because the study has no medical relevance for individual survey participants (no medical research involving human subjects is being conducted) an ethics approval was not compulsory. In terms of data protection and informed consent the study was approved by The Federal Commissioner for Data Protection and Freedom of Information. Verbal informed consent was provided by all participants prior to the interview.

Between September 2009 and July 2010, 22,050 individuals aged 18 years and older were surveyed at one particular point in time. The corresponding cooperation rate at the individual level was 55.8% which represents the cooperation rate at the respondent level [31]. The present analysis is restricted to participants with current physician-diagnosed asthma, defined as an affirmative response to two consecutive questions about whether they had ever been told by a physician that they had asthma and whether it had been present within the past 12 months.

### Data collection

Information on health status, medical history, health-related behaviour, socio-demographic and anthropometric variables was collected based on computer-assisted telephone interviews (CATI). Interviews were performed by interviewers, who were trained and supervised according to guidelines of the Behavioral Risk Factor Surveillance System of the Centers for Disease Control and Prevention, applying a highly standardized protocol [32].

Among participants with current asthma, information on the age of asthma onset, asthma-related health-care utilization (hospitalizations and emergency department admissions) and the history of concomitant conditions (nasal allergy including hay fever, GERD-like symptoms (heartburn or acid regurgitation), intolerance to analgesics, and nasal polyps) was assessed and the asthma outcome was defined as any vs. no exacerbation requiring asthma-related hospitalizations or emergency department (ED) admission in the past year. Although it would be ideal to include information on the application of systemic corticosteroids in the definition of an asthma exacerbation [27], our data permit an approach to define a moderate asthma exacerbation according to the American Thoracic Society and European Respiratory Society [27].

Body mass index (BMI) was calculated by dividing self-reported weight (kg) by height (m) squared. According to the criteria of the World Health Organization, BMI status was categorised as obese (BMI ≥30 kg/m^2^), overweight (25 kg/m^2^ ≤ BMI <30 kg/m^2^) and non-overweight/non-obese (BMI <25 kg/m^2^) [33]. Educational attainment was classified as primary, middle or high according to the 3-level ISCED (International Standard Classification of Education) [34]. Smoking status was classified into three categories (current, former, and never) [35]. Based on the respective response to the question ‘Do you smoke – even occasionally?’ [36], study participants who responded ‘yes, daily’ or ‘yes, occasionally’ were defined as ‘current’ smokers, those who answered ‘no, I quit smoking’ were defined as former and those who answered ‘no, I never smoked’ were defined as ‘never’ smokers.

### Statistical analyses

Data analysis was performed using SPSS (Statistical Package for the Social Sciences) software (version 20, SPSS Inc. Chicago, IL) with the complex sample module. A significance level of *p* < 0.05 was considered statistically significant based on two-tailed tests. In order to assure representativeness at the population level, all results were weighted throughout the analyses and reported along with unweighted numbers of participants. Sample weights adjust for sampling probabilities and selective participation (adjusting for deviations between the study population and German population statistics of December 31, 2008 within strata of age, sex, residential region, and level of education) [31,37]. For each variable, weighted proportions are reported along with the unweighted n referring to the number of participants who provided the information. Weighted prevalence of AR, GERS and AERD as independent study variables and of the outcome variable (asthma outcome as defined by a hospitalization and/or ED admission in the past 12 months) was estimated with a 95% confidence interval (CI). Weighted bivariate analyses were performed to assess the association between asthma comorbidities and covariables (sex, age groups, BMI status, educational attainment, smoking status and asthma duration) and the Rao-Scott chi-square test of independence with second order adjustment was used to test for differences in the distribution of proportions [38]. Independent associations of asthma comorbidities with asthma outcome were examined in multivariable logistic regression models. Sex, chronological age group (18–29, 30–44, 45–64, ≥65 years), BMI status (non-overweight/non-obese, overweight, obese), educational attainment (primary, middle, high), and smoking status (current, former, never) were included as categorical covariables, and asthma duration in years as a continuous covariable.

## Results

### Characteristics of the study population

Basic characteristics of the total study population and of participants with current asthma are summarized in Table 1. Altogether, 1,136 (5.3%) study participants (737 women, 399 men) of a total sample of 22,050 persons aged 18 years and older were included in the present analysis. Their median age at the time of the survey was 52.0 (interquartile range (IQR) 38.0–68.0) years which was higher than the median age of the total study population (48.0; IQR 35.0-64.0). Comparing participants with current asthma to the total study population, higher proportions were found among those who were women, overweight or obese, former smokers and among those with middle or primary educational attainment. Among survey participants with current asthma, those who were women, non-overweight/non-obese, never smokers, or those with middle educational attainment were the most numerous. In addition, almost a quarter of individuals with current asthma were obese. The median asthma duration at the time of the survey was 15.0 (IQR 6.0–28.0) years.

**Table 1:** Descriptive characteristics of study participants of the German National Health Telephone Interview Survey (GEDA) 2010

**Characteristics**	**Current asthma population**	**Total population**
	**(N = 1136)**	**(N = 22050)**
**Sex**, % (n)		
Women	61.1 (737)	51.5 (12483)
**Age**, in years		
Mean (±SD)	52.1 (±18.2)	49.0 (±17.9)
Range	(18–95)	(18–99)
**Age groups**, % (n)		
18–29 years	13.4 (171)	16.9 (3831)
30–44 years	21.5 (276)	25.9 (6096)
45–64 years	35.3 (440)	32.7 (7980)
≥65 years	29.8 (249)	24.4 (4143)
**BMI status**, % (n)		
<25 kg/m^2^	39.3 (488)	47.8 (11322)
25 – <30 kg/m^2^	36.7 (387)	36.3 (7338)
≥30 kg/m^2^	24.0 (237)	15.8 (2931)
**Educational attainment**,% (n)		
Primary	27.1 (126)	22.1 (2082)
Middle	55.7 (625)	55.5 (11076)
High	17.3 (384)	22.3 (8865)
**Smoking status**, % (n)		
Current	29.7 (322)	30.0 (6291)
Former	31.0 (338)	26.6 (5881)
Never	39.3 (476)	43.4 (9873)

Weighted percentage (%), unweighted n may not add up to total N due to unknown or missing responses excluded from the analysis.

### Prevalence of asthma-related comorbidities and asthma outcome among adults with current asthma

Altogether, 75.7% of adults with current asthma reported at least one out of three comorbidities: current allergic rhinitis (AR), current GERD-like symptoms (GERS) or a history of AERD. Reported by almost half of the study participants, GERS was more prevalent than AR among persons with asthma (Table 2). A history of nasal polyps and AERD were also common conditions. In combination with asthma, these conditions are known as Samter’s triad [21]. The study outcome measure – at least one asthma-related hospitalization and/or emergency department admission in the past year – was reported by 9.0% of the survey participants with current asthma.

**Table 2:** Comorbid conditions and study outcome among study participants with current asthma (N = 1136)

**Characteristics**	**Unweighted n**	**Weighted % (95%-CI)**
**Comorbid conditions**		
History of allergic rhinitis	678	54.5 (50.6-58.3)
Current allergic rhinitis (in the past year)	535	42.3 (38.6-46.0)
Current GERD-like symptoms (in the past year)	522	49.6 (45.8-53.4)
History of AERD	138	14.0 (11.3-17.2)
History of nasal polyps	350	29.8 (26.5-33.4)
- among AERD cases	57	36.9 (26.9-48.2)
- among non-AERD cases	287	28.3 (24.8-32.1)
History of AERD and nasal polyps (Samter’s triad)	57	5.2 (3.7-7.3)
**Study outcome**		
Any vs. no asthma-related hospitalization/emergency department admission in the past year	96	9.0 (7.1-11.5)

Weighted prevalence (%) with 95% confidence interval (95%-CI), unweighted count (n). Unknown or missing responses excluded from the analyses. % (n) of missing values for outcome study variable are as follows: any asthma-related hospitalization/emergency department admission in the past year 0.1% (n = 2) missing values. % (n) of missing values for primary independent study variables are as follows: current AR 1.1% (n = 15), current GERS 0.3% (n = 6), and AERD 1.2% (n = 12). Gastroesophageal reflux disease (GERD); acetylsalicylic acid exacerbated respiratory disease (AERD).

### Relationship between comorbidities and socio-demographic and other correlates among adults with current asthma

Statistically significant differences in the distribution of current AR were found according to strata of all covariates but sex (Table 3). Current AR was less prevalent in relation to increasing age group and BMI level whereas it was more prevalent in relation to increasing educational attainment level. The distribution of current AR differed significantly according to smoking status with the highest prevalence among never smokers and the lowest prevalence among former smokers. GERS was significantly more prevalent in relation to increasing BMI level. The prevalence of AERD did not show any significant differences according to covariables.

**Table 3:** Comorbid conditions with respect to covariables among study participants with current asthma (N = 1136)

**Characteristics**	**Current allergic rhinitis**	**Current GERS**	**AERD**
	**(yes vs. no)**	**(yes vs. no)**	**(yes vs. no)**
**Total, %** (n)	42.3 (535)	49.6 (522)	14.0 (138)
**Age,** y			
Mean (±SD)	43.8 ±16.4	52.2 ±17.3	53.2 ±16.6
**Age, %** (n)			
18–29 years	68.2 (113)***	42.1 (72)	6.6 (15)
30–44 years	64.5 (185)***	52.6 (132)	18.4 (38)
45–64 years	39.7 (188)***	51.8 (203)	14.4 (51)
≥65 years	17.8 (49)***	48.3 (115)	13.8 (34)
**Sex, %** (n)			
Men	39.0 (180)	47.2 (181)	13.6 (41)
Women	44.4 (355)	51.2 (341)	14.3 (97)
**BMI status, %** (n)			
<25 kg/m^2^	51.9 (275)***	40.0 (184)**	9.9 (48)
25 – <30 kg/m^2^	40.9 (161)***	53.6 (195)**	16.1 (54)
≥30 kg/m^2^	29.2 (86)***	57.8 (129)**	17.2 (31)
**Educational attainment, %** (n)			
Primary	33.7 (52)**	55.6 (62)	19.6 (20)
Middle	42.4 (282)**	48.2 (296)	12.5 (78)
High	55.8 (201)**	45.3 (164)	10.4 (40)
**Smoking status, %** (n)			
Current	42.3 (140)***	54.6 (170)	12.4 (37)
Former	30.8 (127)***	45.9 (145)	13.0 (42)
Never	51.3 (268)***	48.8 (207)	16.0 (59)
**Asthma duration**, y			
Mean (±SD)	18.4 ±14.1	20.2 ±17.3	18.5 ±16.7

*** p < 0.001, ** p < 0.01, * p < 0.05 obtained from Rao-Scott chi-square test of independence for categorical variables. Unknown or missing responses were excluded from analysis. Unweighted n may not add up to total N due to unknown or missing responses excluded from the analysis. Gastroesophageal reflux disease-like symptoms (GERS); acetylsalicylic acid exacerbated respiratory disease (AERD).

### Association between asthma comorbidities and asthma outcomes

**Figure 1:**
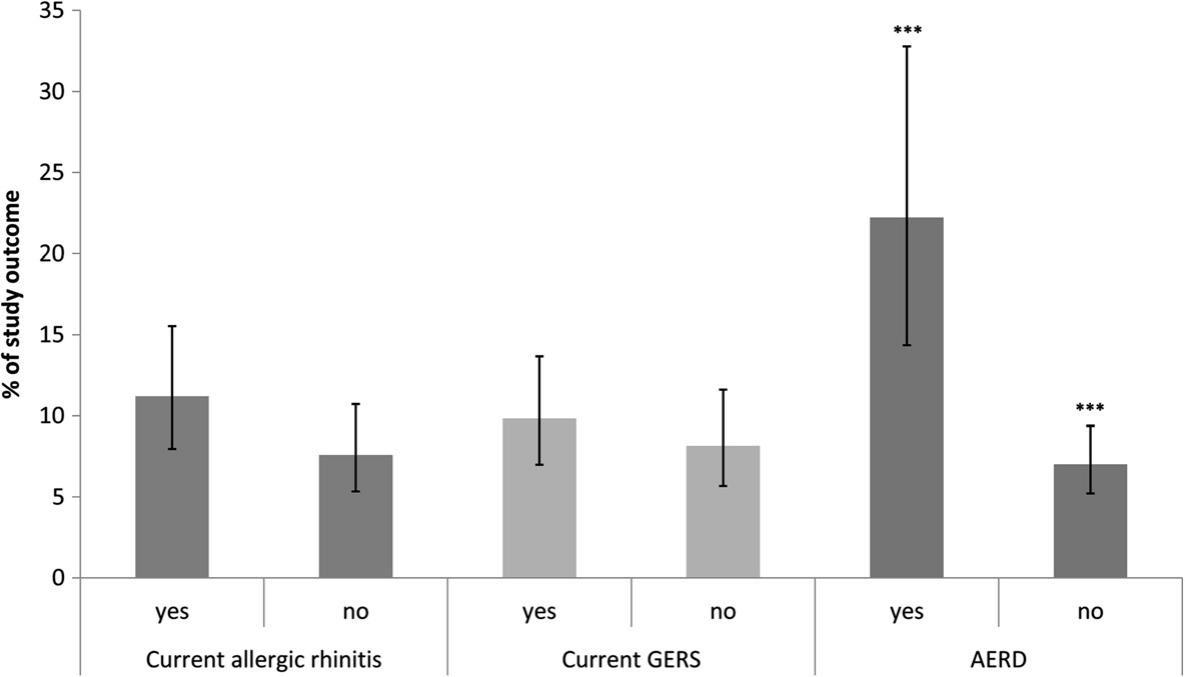
**Asthma-related hospitalizations/emergency department admissions in the past year with regard to comorbidities.** Weighted prevalence (%) of any vs. no asthma-related hospitalization and/or emergency department admissions in the past year among study participants with asthma with regard to concomitant comorbid conditions. *** p < 0.001 obtained from Rao-Scott chi-square test of independence with second order adjustment. Gastroesophageal reflux disease-like symptoms (GERS); acetylsalicylic acid exacerbated respiratory disease (AERD).

The prevalence of at least one asthma-related hospitalization and/or emergency department admission in the past year was higher among asthmatic participants with comorbidities compared to those without (9.8% vs. 8.2% for GERS; 11.2% vs. 7.6% for AR, and 22.2% vs. 7.0% for AERD), but only differences in association with AERD were statistically significant (Figure 1). These results persisted in multivariable models adjusting for covariables (Table 4). An interaction term for sex with AERD additionally included in the multivariable model was non-significant (Additional file 1). There was also no interaction with age group (<55 years vs. ≥55 years) (Additional file 2). The above results also persisted when treating age as a continuous variable in the models (data not shown).

**Table 4:** Association of each comorbidity with any vs. no asthma-related hospitalization/ED admission in the past year

**Any vs. no asthma-related hospitalization/ED admission in the past year**	**Current allergic rhinitis**	**Current GERS**	**AERD**
	**(yes vs. no)**	**(yes vs. no)**	**(yes vs. no)**
	**OR (95%-CI)**	**OR (95%-CI)**	**OR (95%-CI)**
**Model 1**	n = 1120	n = 1128	n = 1122
no	1.00	1.00	1.00
yes	1.53 (0.90–2.62)	1.23 (0.72–2.10)	**3.78 (2.03–7.04)**
**Model 2**	n = 1120	n = 1128	n = 1122
no	1.00	1.00	1.00
yes	1.41 (0.83–2.40)	1.26 (0.73–2.17)	**4.06 (2.20–7.47)**
**Model 3**	n = 1044	n = 1051	n = 1045
no	1.00	1.00	1.00
yes	1.30 (0.75–2.26)	1.12 (0.64–1.98)	**4.49 (2.47–8.16)**

Odds ratio (OR) with 95% confidence interval (95%-CI) obtained from logistic regression models.

*Model 1, unadjusted; model 2, adjusted for age group (18–29, 30–44, 45–64, ≥65) and sex; model 3, adjusted for age group (18–29, 30–44, 45–64, ≥65), sex, educational attainment, smoking status, BMI status, and asthma duration. Gastroesophageal reflux disease-like symptoms (GERS); acetylsalicylic acid exacerbated respiratory disease (AERD).

## Discussion

### Main findings

We found that more than three quarters of adults with asthma reported at least one of the assessed comorbid conditions that are considered highly relevant for asthma outcomes [5]. In the present analysis, every seventh adult with asthma met the criteria of AERD, a condition that by more than four times increased the chance of at least one asthma-related hospitalization or ED admission in the past year. In contrast, we observed an only weak and non-significant association of GERS and AR with the study outcome at the population level.

### Comparison with other studies

In our study, we estimated a life time prevalence of self-reported allergic rhinitis of 54.5% among study participants with asthma. This prevalence estimate is similar to findings of the German national health interview and examination survey (GNHIES) 1998 (52% among individuals with asthma) [39]. Our result is also in line with estimates ranging from 28% (1 year prevalence) up to 100% (life time prevalence) as reviewed for European adults with asthma [26]. Cardinal symptoms of GERD (heartburn or acid regurgitation) were currently present among 49.6% of all study participants with asthma. A systematic review showed similar prevalence estimates of GERD symptoms ranging between 45.0% and 71.0% [16]. Finally, we estimated an AERD prevalence of 14.0% among individuals with asthma which is in the range of reported AIA prevalence varying from 1.9% to 44% among individuals with asthma [24,25,40,41]. In line with our results, AIA prevalence estimates of up to 13.6% were obtained from other population-based surveys among unselected adult asthma populations in Europe and Australia [24,40-42].

Our results regarding the impact of AERD on asthma-specific hospitalizations and/or ED admission are in line with the concept of a specific asthma phenotype. This phenotype has been characterized by frequent exacerbation of symptoms and an increased risk of progressive airflow obstruction compared to ASA-tolerant persons with asthma in a selected patient study [20,22]. In this study, significant differences between the two cohorts were also found with respect to emergency department admissions among individuals with AERD (18%; n = 81) compared to those without (13%; n = 372, p = 0.017) [22]. Individuals with AERD also showed a higher proportion of hospitalizations: 6% (n = 29) vs. 5% (n = 131), p = 0.068 [22]. Likewise in a case comparison with community control, the odds ratio of an hospitalization for acute asthma was more than three times increased for ASA/NSAID-intolerant asthma patients compared to ASA/NSAID-tolerant asthma patients (OR = 3.63; 95%-CI = 1.70-7.74) [23]. Additionally in a population-based study, significant differences were found with respect to the prevalence of an asthma-specific emergency department admission: 28.8% (n = 52) among individuals with AIA vs. 12.2% (n = 135) among subjects with ASA-tolerant asthma and with respect to an asthma-specific hospitalization: 15.0% (n = 27) vs. 6.2% (n = 69) [24].

We cannot exclude the possibility that aspects of self-management such as good medication adherence or proper inhalation technique [43] influenced the asthma outcome. Moreover, discordant comorbidity such as mental disorders [44] may have contributed but this was beyond the scope of the present analysis. However, worse outcomes among persons with AERD may, in part, also have been modifiable by effective therapeutic interventions such as ASA desensitization followed by daily ASA ingestion [19] or treatment with leukotriene modifiers [6,20], contributing to the integral management of this complex disease. Given that AERD constitutes a considerable part of the general adult asthma population, future clinical and epidemiological research is thus needed to understand whether AERD populations are adequately managed and to what extent disease outcomes might be modifiable by optimal care.

We found that at the population-based level the presence of cardinal symptoms of GERD did not result in a significantly higher chance of at least one asthma-related hospitalization or ED admission in the past year. It has previously been hypothesized that GERD may act as a potential asthma trigger through various mechanisms by increasing airway inflammation and airway responsiveness [17]. Treatment studies, however, only inconsistently suggested an association between GERD and asthma outcomes [17]. Moreover, asthma exacerbation outcome was not significantly affected by long-term proton-pump inhibitor (PPI) treatment among asthmatics with symptoms suggestive of GERD in a large recent randomized controlled trial (RCT) [18]. In contrast, other authors of a large RCT observed a significant reduction in moderate-to-severe asthma exacerbations due to PPI treatment (4% vs. 13.9%, respectively; p = 0.016) [45].

Moreover, we found that the size of the independent effect of current AR on the study outcome measure was low and non-significant. Both conditions are considered to be linked and to interact on the basis of a common inflammatory process. AR might also affect asthma by other means such as an impairment of the air-conditioning and filtering function of the nose or an increase in mouth breathing due to nasal obstruction, resulting in a higher exposure of the lower airways to airborne asthma triggers [7,46]. In the published literature, concomitant AR appears to have an impact on asthma outcomes; to what extent, however, is still under debate [5,7,8,10,46]. A retrospective observational study based on a general practice database and a post hoc analysis of a clinical trial assessed the association of concurrent AR with asthma outcomes in adults. In both studies moderate ORs for an asthma-related hospitalization (pooled with asthma-related emergency department admissions) (adjusted OR = 1.52; 95%-CI = 1.03–2.24) [12] and on asthma-related emergency department admissions (adjusted OR = 2.32; 95%-CI = 1.12-4.80) [7,11] were reported. In contrast, fewer asthma-related hospitalizations (adjusted p = 0.01) were observed among atopic asthma patients with nasal symptoms (mean number = 0.2) compared to those without (mean number = 0.6) in a retrospective observational study based on medical records of a university-based asthma clinic [7,47]. Moreover, prior literature suggests that treatment of concomitant AR in persons with asthma can influence asthma outcomes and reduce asthma exacerbations [7,8,10]. The recognition and consequent management of AR in asthma has thus been promoted by national and supranational guidelines during recent years [6,14,48,49]. Therefore, the insignificant effect of AR on the study outcome might reflect improved clinical management of AR within the general asthma population.

### Limitations and strengths

The present study was of cross-sectional design and information on the presence of current asthma and related comorbidities was based on telephone interviews. Hence, some limitations need to be discussed. First, our case definition of current asthma was based on an interview questionnaire and did not include objective measurements. However, Kilpeläinen et al. showed that a questionnaire-based definition of physician-diagnosed asthma had acceptable levels of positive predictive value and specificity and can be considered a suitable method of assessment in epidemiological studies [50]. Second, we cannot rule out misclassification with respect to AR and AERD. Regarding the history of AERD, we must consider that the gold standard for the diagnosis of AERD in clinical studies is a graded ASA provocation test [20] whereas a questionnaire-based assessment of the patient’s history would reveal lower prevalence estimates than the objective assessment based on oral provocation testing [25]. In large population-based studies, self-reported information is often the only feasible option. Notwithstanding these limitations, our prevalence estimates of concomitant current AR and of AERD as well as of cardinal symptoms of GERD lie in the range of results obtained on the basis of questionnaires discussed in recent reviews [16,25,26].

Third, we could not perform non-response analyses based on information collected for non-responders and responders. However, all data were carefully weighted to adjust for sampling design and non-response in order to represent the adult residential population of Germany and to extend the generalizability of our results to the general population. Finally, the cross-sectional study design does not allow conclusions to be reached regarding the cause-effect relationship(s) of comorbidity in asthma patients; therefore, our results need to be interpreted with caution.

The major strength of the study is that our analyses were based on a large population-based sample of the German adult population, allowing us to quantify the impact of major comorbid conditions in a general asthma population. Our study adds to the limited knowledge on specific comorbidity asthma outcome relations throughout adult life. We have been able to show that AERD is a common condition and contributes considerably to increased disease-specific hospital contacts in the general asthma population. Our findings are thus of interest for both clinicians in primary and specialist care. In addition, our data underline the socio-economic impact of asthma with complicating AERD and are relevant for the organization of health care.

## Conclusions

Three quarters of German adults with asthma had at least one of the 3 following comorbidities GERS, AR, AERD. Our results underline the public health relevance and importance of recognizing major comorbidities in asthma care. Particularly AERD contributes considerably to hospital contacts in the asthma population. Further clinical and epidemiological research is needed to determine whether the clinical management of specific asthma populations is consistent with current recommendations and to what extent disease outcomes might be improved by optimal care.

## Abbreviations

AERD: Acetylsalicylic acid exacerbated respiratory disease; AIA: Acetylsalicylic acid-induced asthma; AR: Allergic rhinitis; ASA: Acetylsalicylic acid; BMI: Body mass index; CATI: Computer-assisted telephone interviews; CI: Confidence interval; ED: Emergency department; GEDA: German National Health Telephone Interview Survey; GERD: Gastroesophageal reflux disease; GERS: Gastroesophageal reflux disease-like symptoms; IQR: Interquartile range; ISCED: International Standard Classification of Education; NSAID: Nonsteroidal anti-inflammatory drugs; OR: Odds ratio; PPI: Proton-pump inhibitor; RCT: Randomized controlled trial; SPSS: Statistical Package for the Social Sciences.

## Competing interests

The authors declare that they have no competing interest.

## Authors’ contributions

HS, UL, CS participated in the concept and design of the study. All authors participated in the design of the analysis plan and the interpretation of the data. HS, UL, CS analysed the data and drafted the manuscript. UL, CS, and TK critically revised the manuscript and supervised HS. All authors read and approved the final manuscript.

## Pre-publication history

The pre-publication history for this paper can be accessed here:

http://www.biomedcentral.com/1471-2466/13/46/prepub

## Supplementary Material

Additional file 1: Table S4a Sex-specific association of comorbidities with any vs. no asthma-related hospitalization/ED admission in the past year. Sex-specific odds ratio (OR) with 95% confidence interval (95%-CI) obtained from logistic regression models. *Model 4, adjusted for age group (18–29, 30–44, 45–64, ≥65), educational attainment, smoking status, BMI status, and asthma duration; model 5, adjusted for age group (18–29, 30–44, 45–64, ≥65), sex, educational attainment, smoking status, and BMI status, asthma duration plus sex*current allergic rhinitis/current GERS/AERD. Gastroesophageal reflux disease-like symptoms (GERS); acetylsalicylic acid exacerbated respiratory disease (AERD). Click here for file

Additional file 2: Table S4b Age-specific association of comorbidities with any vs. no asthma-related hospitalization/ED admission in the past year. Age-specific odds ratio (OR) with 95% confidence interval (95%-CI) obtained from logistic regression models. *Model 6, adjusted for age group (18–29, 30–44, 45–64, ≥65), sex, educational attainment, smoking status, and BMI status, asthma duration; model 7, adjusted for age group (≥55 vs. <55), age, sex, educational attainment, smoking status, and BMI status, asthma duration plus age group (≥55 vs. <55)*current allergic rhinitis/current GERS/AERD. Gastroesophageal reflux disease-like symptoms (GERS), and acetylsalicylic acid exacerbated respiratory disease (AERD). Click here for file
