# Supplementary material for: Major comorbid conditions in asthma and association with asthma-related hospitalizations and emergency department admissions in adults: results from the German national health telephone interview survey (GEDA) 2010
Source: BMC Pulm Med. 2013 Jul 12;13:46. doi: 10.1186/1471-2466-13-46 (PMC3718654; doi:10.1186/1471-2466-13-46)
Supplement: Additional file 1: Table S4a — Sex-specific association of comorbidities with any vs. no asthma-related hospitalization/ED admission in the past year. Sex-specific odds ratio (OR) with 95% confidence interval (95%-CI) obtained from logistic regression models. *Model 4, adjusted for age group (18–29, 30–44, 45–64, ≥65), educational attainment, smoking status, BMI status, and asthma duration; model 5, adjusted for age group (18–29, 30–44, 45–64, ≥65), sex, educational attainment, smoking status, and BMI status, asthma duration plus sex*current allergic rhinitis/current GERS/AERD. Gastroesophageal reflux disease-like symptoms (GERS); acetylsalicylic acid exacerbated respiratory disease (AERD). [file 1471-2466-13-46-S1.pdf]

Additional Table 4.a - Sex-specific association of comorbidities with any vs. no asthma-related hospitalization/ED admission in the past year.

**Any vs. no asthma-related hospitalization/ED admission in the past year**		**Current allergic rhinitis** (yes vs. no) OR (95%-CI)	**Current GERS** (yes vs. no) OR (95%-CI)	**AERD** (yes vs. no) OR (95%-CI)
**Men**	**Model 4**			
	no	1.00	1.00	1.00
	yes	1.05 (0.41–2.66)	0.71 (0.26–1.92)	4.37 (1.64–11.64)
**Women**	**Model 4**			
	no	1.00	1.00	1.00
	yes	1.68 (0.83–3.42)	1.50 (0.81–2.80)	4.71 (2.27–9.78)
**Model 5**				
Interaction term sex*comorbid condition		0.14	0.24 0.74	

Odds ratio (OR) with 95% confidence interval (95%-CI) obtained from logistic regression models. *Model 4, adjusted for age group (18–29, 30–44, 45–64, Ó65), educational attainment, smoking status, BMI status, and asthma duration; model 5, adjusted for age group (18–29, 30–44, 45–64, ≥65), sex, educational attainment, smoking status, and BMI status, asthma duration plus sex*current allergic rhinitis/current GERS/AERD. Gastroesophageal reflux disease-like symptoms (GERS); acetylsalicylic acid exacerbated respiratory disease (AERD).
