# Supplementary material for: Major comorbid conditions in asthma and association with asthma-related hospitalizations and emergency department admissions in adults: results from the German national health telephone interview survey (GEDA) 2010
Source: BMC Pulm Med. 2013 Jul 12;13:46. doi: 10.1186/1471-2466-13-46 (PMC3718654; doi:10.1186/1471-2466-13-46)
Supplement: Additional file 2: Table S4b — Age-specific association of comorbidities with any vs. no asthma-related hospitalization/ED admission in the past year. Age-specific odds ratio (OR) with 95% confidence interval (95%-CI) obtained from logistic regression models. *Model 6, adjusted for age group (18–29, 30–44, 45–64, ≥65), sex, educational attainment, smoking status, and BMI status, asthma duration; model 7, adjusted for age group (≥55 vs. <55), age, sex, educational attainment, smoking status, and BMI status, asthma duration plus age group (≥55 vs. <55)*current allergic rhinitis/current GERS/AERD. Gastroesophageal reflux disease-like symptoms (GERS), and acetylsalicylic acid exacerbated respiratory disease (AERD). [file 1471-2466-13-46-S2.pdf]

Additional Table 4.b - Age-specific association of comorbidities with any vs. no asthma-related hospitalization/ED admission in the past year.

**Any vs. no asthma-related hospitalization/ED admission in the past year**		**Current allergic rhinitis** (yes vs. no) OR (95%-CI)	**Current GERS** (yes vs. no) OR (95%-CI)	**AERD** (yes vs. no) OR (95%-CI)
**Age <55 years**	**Model 6**			
	no	1.00	1.00	1.00
	yes	1.72 (0.81–3.67)	1.10 (0.55–2.20)	4.69 (2.18–10.10)
**Age ≥55 years**	**Model 6**			
	no	1.00	1.00	1.00
	yes	0.68 (0.19–2.46)	1.55 (0.60–3.97)	4.56 (1.66–12.54)
**Model 7**				
Interaction term age group (≥55 vs. <55)*comorbid condition		0.07	0.64	0.83

Odds ratio (OR) with 95% confidence interval (95%-CI) obtained from logistic regression models. *Model 6, adjusted for age group (18–29, 30–44, 45–64, ≥65), sex, educational attainment, smoking status, and BMI status, asthma duration; model 7, adjusted for age group (≥55 vs. <55), age, sex, educational attainment, smoking status, and BMI status, asthma duration plus age group (≥55 vs. <55)*current allergic rhinitis/current GERS/AERD. Gastroesophageal reflux disease-like symptoms (GERS), and acetylsalicylic acid exacerbated respiratory disease (AERD).
